# Therapy with Interleukin-22 Alleviates Hepatic Injury and Hemostasis Dysregulation in Rat Model of Acute Liver Failure

**DOI:** 10.1155/2014/705290

**Published:** 2014-04-01

**Authors:** Tariq Helal Ashour

**Affiliations:** Department of Laboratory Medicine, Faculty of Applied Medical Sciences, Umm Al-Qura University, P.O. Box 7607, Makkah 7152, Saudi Arabia

## Abstract

The therapeutic efficacy of interleukin-22 (IL-22) on liver injury and hematological disturbances was studied in rat model of acute liver failure (ALF) induced by D-galactosamine/lipopolysaccharide (D-GalN/LPS). The following parameters were investigated: (1) survival rate, (2) serum levels of liver function enzymes (aspartate aminotransferase (AST), alanine aminotransferase (ALT), and alkaline phosphatase (ALP)), total bilirubin (TBILI), and total albumen (ALB), (3) blood clotting tests (prothrombin time (PT), activated partial thromboplastin time (aPTT), and fibrinogen level (FIB)) and white blood cells (WBCs), red blood cells (RBCs), and platelet counts, (4) hepatic levels of tumor necrosis factor-**α** (TNF-**α**) and cyclooxygenase-2 (COX-2), and (5) liver histopathology. After 48 hours of D-GalN/LPS, the rats exhibited 20% mortality, significant increases in AST, ALT, ALP, TBILI, PT, and aPTT, TNF-**α**, and COX-2 and significant decreases in FIB, WBCs, and RBCs. By contrast, therapy with IL-22 prevented the lethal effect of D-GalN/LPS by 100% and efficiently alleviated all the biochemical and hematological abnormalities that were observed in ALF untreated group. Furthermore, IL-22 treatment decreased the hepatic contents of TNF-**α** and COX-2. The histopathological findings also supported the hepatoprotective effect of IL-22. Taken together, therapy with IL-22 can represent a promising therapeutic tool against liver injury and its associated hemostasis disturbances.

## 1. Introduction

Acute liver failure (ALF) and fulminant hepatitis (FH) are devastating liver diseases with multiple etiologies, coagulability dysfunctions, poor prognosis, and 90% overall mortality rate [1]. Up till now there are no effective treatment therapies for this disease and its highly fatal complications [2]. Coherently, development of more effective and highly selective therapeutic strategy is a paramount medical need.

IL-22 is a newly emerged cytokine with unique biological activities. Various cell types of hematopoietic origin produce IL-22, such as innate lymphoid cells, NK, NKT, and *γδ* T cells [3]. IL-22 exerts its biological functions via activation of membrane-bound heterodimeric receptor complex consisting of IL-22R1 and IL-10R2, which is predominantly expressed by epithelial cells including gut and liver cells [4, 5]. In addition to its defense role against infectious pathogens, numerous studies demonstrated the favorable tissue protective properties of either exogenously administered or endogenously overexpressed IL-22. In this concept, hepatocytes abundantly express IL-22R and its stimulation via IL-22 promotes hepatocyte growth and survival [5].

The hepatoprotective effects of IL-22 have recently been suggested in a variety of hepatocellular damages [6–9]. For example, IL-22 promotes liver regeneration after hepatectomy [7], protects against acute alcohol-induced hepatotoxicity [8], and ameliorates liver fibrogenesis via induction of the senescence of hepatic stellate cell [9], suggesting a therapeutic implication of this cytokine in liver transplantation or patients undergoing hepatic surgery. IL-22 has been shown to promote proliferation of liver stem/progenitor cells in mice and patients with chronic HBV infection [10]. Furthermore, an inverse correlation between the degree of liver fibrosis and IL-22 concentration was recently detected in the liver tissues of patients with chronic HBV infection [11].

Based on these observations, an application of exogenous IL-22 or induction of its endogenous production may represent an innovative therapeutic option in human patients with acute or chronic liver disease [5, 11, 12]. Therefore, the present study was designed to investigate the therapy efficacy or IL-22 therapy against ALF and its associated hemostasis and hematological alterations that are induced rats by D-GalN/LPS.

## 2. Materials and Methods

### 2.1. Chemicals and Reagents

Recombinant rat interleukin-22 (rIL-22) was purchased from R&D Systems (Minneapolis, MN, USA). Commercial enzyme-linked immunosorbent assay (ELISA) kits of rat COX-2 rat TNF-*α* were purchased from R&D Systems and IBL International (GmbH, Hamburg, Germany), respectively. D-Galactosamine (D-GalN) and phenol extracted lipopolysaccharide from* Escherichia coli* (LPS) were obtained from Sigma-Aldrich (St. Louis, MO, USA). Other used chemicals and reagents are stated under the sections of their applications.

### 2.2. Animals and Experimental Approach

All experimental protocols were approved by the Committee for the Care and Use of Laboratory Animals at Umm Al-Qura University and were in accordance with the Guide for the Care and Use of Laboratory Animals published by the U.S. National Institutes of Health [13].

In this study, thirty adult male Wistar rats weighing 230 ± 15 g were randomly and equally assigned into 3 groups: group I; control rats did not receive any treatment; group II; D-GalN/LPS group in which rats were intraperitoneally (i.p.) injected with a nonhighly lethal dose of D-GalN (400 mg/kg BW) and LPS (40 *μ*g/kg BW) dissolved in 1 mL of sterile saline as described previously [14]; and group III: D-GalN/LPS + rIL-22 group in which the rats were treated with two doses of rIL-22 (1 *μ*g/g BW/dose, dissolved in 0.5 mL saline; i.p.) at time 0 and 6 hrs after D-GalN/LPS injection, respectively.

All animals were observed for 48 hrs for survivability, and two rats from group II only were dead. At the end of the experiments, all animal groups were sacrificed under ether anesthesia and their blood specimens were collected and their livers were harvested for the target examinations.

### 2.3. Blood Sampling and Analysis

During scarification process, three blood samples were immediately withdrawn from the vena cava of each rat and used for blood coagulation, hematology, and biochemical analyses. The first sample was collected in a tube that contained 0.11 M sodium citrate anticoagulant (1 : 9, v : v) and used for plasma preparation for screening of the following blood coagulation tests: prothrombin time (PT), activated partial thromboplastin time (aPTT), and fibrinogen concentrations (FIB), by using Dade Behring reagents and following manufacturer's instructions as previously described [15]. The second sample was collected in a tube that contained disodium salt of ethylene diamine tetra acetic acid (EDTA) anticoagulant and used for determination of the following hematology parameters: erythrocyte count (RBC), leukocyte count (WBC), and platelet count (PLT). The last portion of the collected blood was placed in a plain centrifuge tube without any anticoagulant and used for assessment of the serum concentrations of liver function enzymes (aspartate aminotransferase (AST), alanine aminotransferase (ALT), and alkaline phosphatase (ALP)), albumin (ALB), and total bilirubin (TBILI) using commercially available diagnostic kits (Biomerieux SA, France), and according to manufacturer's instructions.

### 2.4. ELISA Assays of TNF-*α* and COX-2 Concentrations in Liver

After blood withdrawal, the livers were harvested quickly, and a portion of each isolated liver was homogenized in RIPA lysis buffer (1 : 6, w : v) and then centrifuged at 10,000 rpm for 10 min at 4°C. The obtained supernatant was used for measurement the intrahepatic concentrations of TNF-*α* and COX-2 proteins by using ELISA kits and an automated ELISA analyzer (HUMAN, Biochemica und Diagnostica, MBH, Germany). All samples were processed in duplicate and according the manufacturer's instructions.

### 2.5. Histological Analysis

For histopathological investigations, blocks of all isolated livers were fixed in 10% buffered formalin, embedded in paraffin, sectioned into 5 *μ*m-thickness slices, stained with hematoxylin and eosin (H&E), and examined with a light microscopy in a blinded fashion for the presence of the hallmarks of hepatic injury.

### 2.6. Statistical Analysis

The results were expressed as the mean ± standard deviation and statistical analysis was carried out using SPSS software, version 16.0 (SPSS Inc., Chicago, IL, USA). One-way analysis of variance (ANOVA) followed by Student's* t*-test was used to analyze the statistical differences. Moreover, the statistical significance of survival rate among the groups was determined by using* Chi-square* test. *P* < 0.05 was considered to represent a statistically significant difference.

## 3. Results

### 3.1. Hepatoprotective Effect of IL-22 Therapy

**Figure 1:**
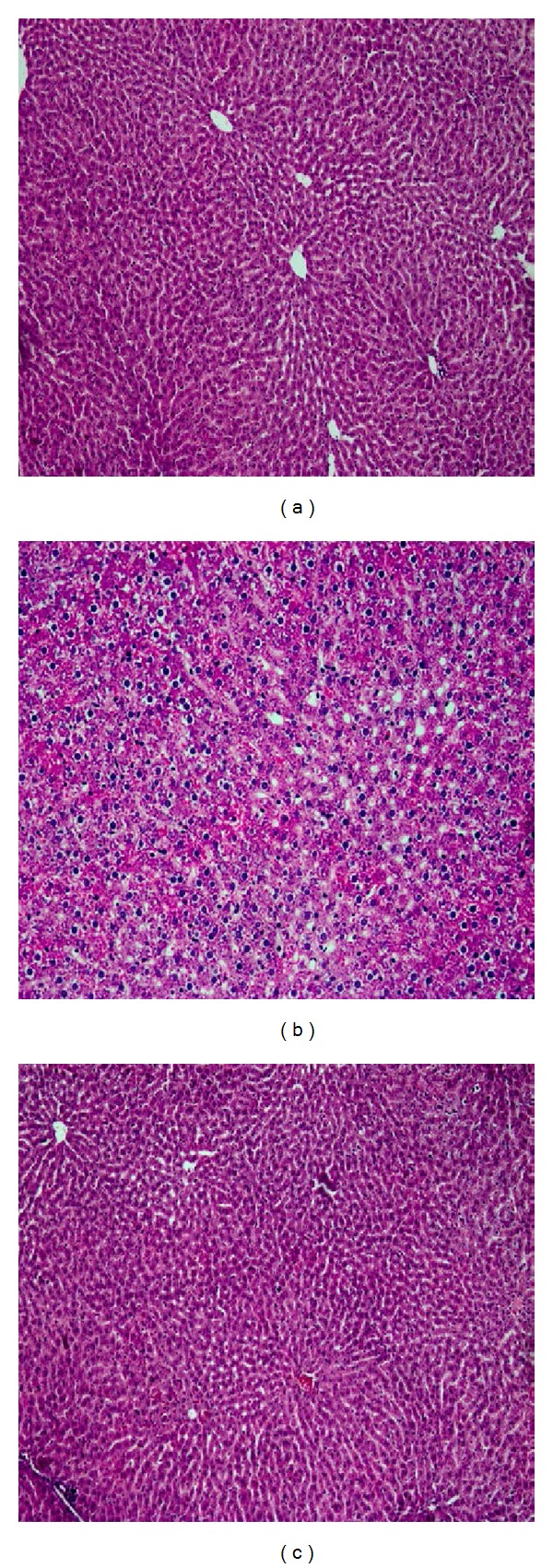
Hepatoprotective effect of IL-22 therapy against D-GalN/LPS-induced acute liver injury in rats. In comparison with livers of normal control rats (a), after 48 h from injection of D-GalN/LPS, there was severe hepatic injury that reflected a high degree of hepatocellular necrosis, apoptosis, and inflammatory cell infiltration (b); however, livers of rats injected with D-GalN/LPS and then treated with IL-22 (c) showed scant pathological foci and undetectable inflammatory cell infiltration.

All animal groups were monitored over 48 h after D-GalN/LPS injection to determine their survival rate. As shown in Table 1, the injected D-GalN (400 mg/kg) plus LPS (40 *μ*g/kg) resulted in 20% mortality rate associated with severe hepatic injury reflected by significant increases in serum levels of liver function enzymes (AST, ALT, and ALP) and TBILI and significant decreases in serum ALB. On the other hand, administration of IL-22 after D-GalN/LPS kept the rats survivability by 100% and markedly protected their livers, as evidenced by returning the serum levels of AST, ALT, ALP, TBILI, and ALB almost near their baseline control values (Table 1). The histopathological findings also supported the biochemical observations. As illustrated in Figure 1, livers of D-GalN/LPS group showed a high degree of hepatocellular necrosis, apoptosis, and inflammatory cell infiltration; however, rats that were injected with D-GalN/LPS and treated with IL-22 showed scant pathological foci and undetectable inflammatory cell infiltration in their lives.

### 3.2. Blood Coagulation Tests and Hematological Findings

**Figure 2:**
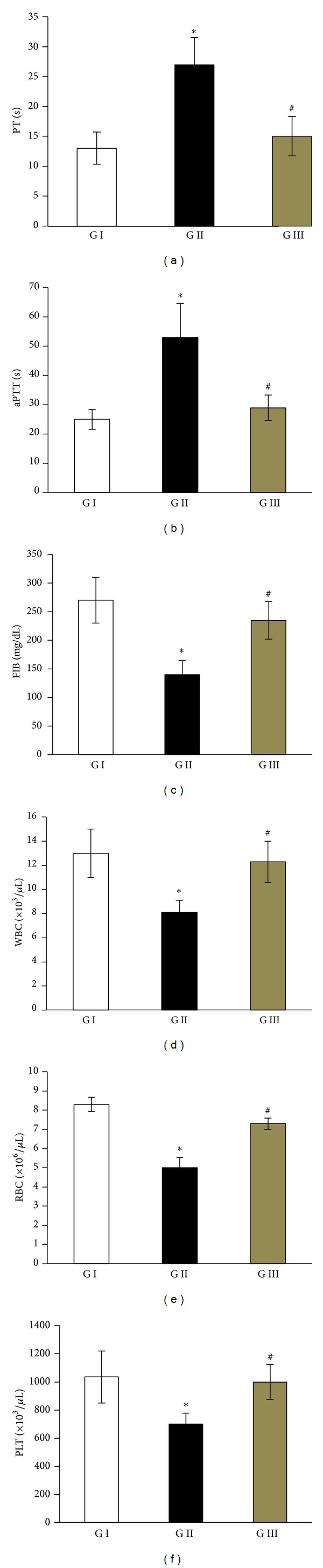
Alleviatived effects of IL-22 therapy on the alterations of blood coagulation tests and blood cell counts in D-GalN/LPS injected rats. GI: control group, GII: D-GalN/LPS group, and GIII: D-GalN/LPS + IL-22 group. Values are represented as mean ± SD. *P < 0.05 versus control group; ^#^ *P* < 0.05 versus D-GalN/LPS untreated group.

Coagulation and hematological abnormalities are common in human patients with acute liver injury. In consistency, rats with acute liver injury caused by D-GalN/LPS showed significant alterations of blood clotting tests: PT, aPTT, and FIB, indicating the influence on the intrinsic, extrinsic, and common pathway of coagulation. As shown in Figure 2, PT and aPTT values were prolonged more than 2 times, and FIB values were reduced to ≥2-folds after 48 h of injection of D-GalN/LPS relative to their control values. Also, as compared with control values, D-GalN/LPS significantly decreased the blood counts of WBCs, RBCs, and PLT (Figure 2). By contrast, treatment of D-GalN/LPS-injected rats with IL-22 had significantly succeeded in reversion of all these abnormalities in blood coagulation tests and counts of WBCs, RBCs, and PLT (Figure 2).

### 3.3. Hepatic Levels of TNF-*α* and COX-2

**Figure 3:**
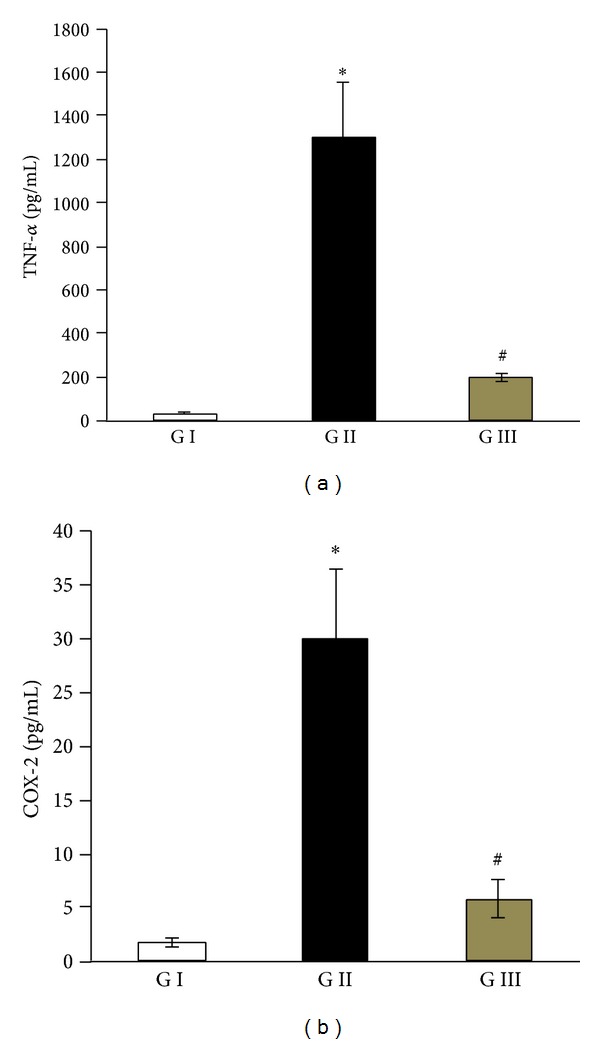
Levels of proinflammatory mediators; tumor necrosis factor-*α* (TNF-*α*), and cyclooxygenase-2 (COX-2), in the liver tissues. GI: control group, GII: D-GalN/LPS group, and GIII: D-GalN/LPS + IL-22 group. Values are represented as mean ± SD. *P < 0.05 versus control group; ^#^ *P* < 0.05 versus D-GalN/LPS untreated group.

There is strong evidence that D-GalN/LPS-induced acute liver injury and fulminant hepatitis are associated with liver infiltration with inflammatory immune cells with subsequent abundant increase in the production of proinflammatory mediators including TNF-*α* and COX-2. To confirm this fact, the concentrations of these two molecules were measured in the livers of all animal groups after 48 h form D-GalN/LPS. As demonstrated in Figure 3, livers of normal control group contain extremely very low or even undetectable levels of TNF-*α* and COX-2; however, after 48 h of D-GalN/LPS injection there was a significant increase in the intrahepatic levels of these two proinflammatory molecules. In contrast, therapy with IL-22 was efficiently succeeded in inhibiting the stimulating effects of D-GalN/LPS on TNF-*α* and COX-2 production in liver tissues (Figure 3).

## 4. Discussion

Despite the substantial advances in controlling of human diseases, the impact of liver diseases is still worldwide a major health problem. Bacterial endotoxin (LPS) derived from intestinal bacteria is implicated in the pathogenesis of several acute and chronic inflammatory liver diseases [1, 16]. At the experimental level, LPS/D-GalN-induced liver injury is a well-established animal model of acute liver failure (ALF) and fulminant hepatitis. In this model D-GalN blocks gene transcription in the liver and LPS in turn induces an acute cytokine-dependent liver inflammation accompanied by massive liver necrosis, hemostasis disturbances, and death of the animals [17–19]. The current study showed that therapy with IL-22 completely prevented the mortality and significantly alleviated the hepatic damage and the deteriorated blood cell counts and coagulation tests in a rat model of ALF induced by D-GalN/LPS. Moreover, it also significantly suppressed the production of TNF-*α* and COX-2 in the liver tissues of rats injected with D-GalN/LPS. Coherently, IL-22 may be a promising therapeutic agent that can reverse the hepatic injury and subsequent coagulation dysfunction in patients with acute hepatitis.

IL-22 is a recently discovered cytokine with pivotal biological activities that its hepatoprotective effects have recently been suggested in a variety of hepatocellular damages [6]. In this concept, it has been postulated that IL-22 is a survival factor for hepatocytes and induces antiapoptotic and mitogenic gene expression in the liver cells [20, 21]. IL-22 was found to enhance liver regeneration [7] and protect the liver against fatty liver disease [22], as well as against ethanol-, concanavalin A-, carbon tetrachloride-, and Fas ligand-induced liver injury [8, 20, 21]. Additionally, an inverse correlation between the degree of liver fibrosis and IL-22 concentration was recently observed in the liver tissues of patients with chronic HBV infection, hypothesizing that IL-22 may also play an antifibrotic role in human liver diseases [9–12]. In agreement with these previous findings, the remarkable hepatoprotective effects of IL-22 against D-GalN/LPS-induced acute liver injury in rats were significantly detected at the biochemical and histopathological levels (Table 1 and Figure 1).

The liver is the major organ for synthesis of blood clotting factors and blood proteins. Consequently, coagulation and hematological abnormalities are common in human patients with acute liver injury [19]. Consistent with this fact, rats injected with D-GalN/LPS had developed severe hepatic injury, as evidenced by elevations in the serum levels of liver function enzymes (AST, ALT, and ALP) and total bilirubin and a significant decrease in total albumen, which were associated with coagulation dysfunction reflected by prolonged PT and aPTT, and decreased fibrinogen levels. Moreover, there was a significant reduction in the blood WBCs, RBCs, and platelet counts in rats received D-GalN/LPS. Similar findings of hemostasis disturbances in D-GalN/LPS-induced ALF have also been previously reported by Korish [19]. Interestingly, therapy with IL-22 after D-GalN/LPS injection had not only reversed the abnormal liver functions and blood coagulation tests but also numbers of WBCs, RBSc, and platelets almost near to their normal control values (Figure 2). In support, Liang et al. [23] revealed that injection of IL-22 into mice modulates the factors involved in coagulation, including fibrinogen levels and platelet numbers, and cellular constituents of blood, such as neutrophil and RBC counts. In their study, mice treated with IL-22 showed significant increases in platelets and neutrophils counts in their blood, and an enhanced fibrinogen transcript in their liver [23]. Collectively, these findings indicate that IL-22 can alleviate the hepatic damage and disturbances in blood hemostasis and cellularity that were observed in acute liver injury as in D-GalN/LPS model.

There is ample evidence of the central pathogenic role of TNF-*α* in development of a variety of liver disease modalities, particularly in mortality, acute liver injury, and fulminant hepatitis induced by D-GalN/LPS [17, 18, 24]. TNF-*α* causing fatal hepatic failure and septic shock in humans has also been reported [25]. Blocking of TNF-*α* synthesis or activity can attenuate LPS-induced liver injury, and this confirms the pivotal role of TNF-*α* in sepsis-related liver toxicity [26, 27]. Interestingly, the results of the current study are in harmony with these facts, whereas rats injected with D-GalN/LPS and left without treatment exhibited abundant release of TNF-*α* in their liver tissues, and this phenomenon was markedly inhibited via IL-222 therapy (Figure 3).

In the present study, D-GalN/LPS significantly induced the hepatic COX-2 production, and treatment of these animals with IL-22 was efficiently succeeded to counteract this effect (Figure 3). In support, Chang and his colleagues demonstrated that therapy with IL-22 directly suppressed the production of COX-2 in injured myocardial cells [28]. COX-2 has a crucial role in the pathogenesis of inflammation by synthesis of potent inflammatory mediators (prostanoids) in inflamed tissues. LPS has been shown to stimulate the expression of COX-2 in various human cells including hepatocytes [29], and intrahepatic expression of COX-2 is induced in liver injury by LPS [30]. More interestingly, to evaluate the effect of hepatocyte COX-2 in D-GalN/LPS-induced liver injury, Han et al. [31] had generated transgenic mice with targeted expression of COX-2 in the liver, and then the animals were injected with D-GalN/LPS. In comparison with wild type mice, the COX-2 transgenic mice exhibited earlier mortality, higher serum ALT and AST levels, and more prominent liver tissue damage. Moreover, pretreatment of these COX-2 transgenic mice with a selective COX-2 inhibitor markedly attenuated D-GalN/LPS-induced liver damage, suggesting that hepatocyte COX-2 and its downstream signaling pathway accelerate LPS-induced liver injury [31].

## 5. Conclusions

The present data indicate that therapy with IL-22 has a potent protective effect against D-GalN/LPS-induced liver injury, coagulation, and hematological disturbances in rats. The beneficial effects of IL-22 could, at least in part, be due to the direct and/or indirect inhibition of proinflammatory factors, such as TNF-*α* and COX-2. These findings can also suggest the potential therapeutic application of IL-22 for the treatment of acute liver damage and its associated coagulation dysfunction.

## Figures and Tables

**Table 1:** Effects of IL-22 therapy on the survival rate and liver function serobiomarkers of rats 48 h after D-GalN/LPS injection.

Groups	AST (IU/L)	ALT (IU/L)	ALP (IU/L)	TBILI (mg/dL)	ALB (g/dL)	Mortality (%)
Control	105 ± 15.1	53.7 ± 8.9	211 ± 39.3	0.08 ± 0.01	4.4 ± 0.9	0
D-GalN/LPS	410 ± 45.8*	172 ± 31*	853 ± 214*	1.1 ± 0.02*	1.7 ± 0.3*	20*
D-GalN/LPS + IL-22	143 ± 23.3^#^	62.6 ± 11.9^#^	259 ± 41^#^	0.1 ± 0.02^#^	4.1 ± 0.7^#^	0^#^

Values are represented as mean ± SD. *P < 0.05 versus control group; ^#^ *P* < 0.05 versus D-GalN/LPS group.
